# The Cooling Effect on Proinflammatory Cytokines Interferon-Gamma, Tumor Necrosis Factor-Alpha, and Nitric Oxide in Patients with Multiple Sclerosis

**DOI:** 10.1155/2013/964572

**Published:** 2013-05-16

**Authors:** Turan Poyraz, Egemen Idiman, Sezer Uysal, Leyla Iyilikci, Serkan Özakbaş, Esra Coskuner Poyraz, Fethi Idiman

**Affiliations:** ^1^Division of Neuroimmunology, Department of Neurology, Faculty of Medicine, Dokuz Eylül University, 35345 İzmir, Turkey; ^2^Department of Biochemistry, Faculty of Medicine, Dokuz Eylül University, 35345 İzmir, Turkey; ^3^Department of Anesthesiology and Reanimation, Faculty of Medicine, Dokuz Eylül University, 35345 İzmir, Turkey

## Abstract

Multiple sclerosis (MS) is the most common inflammatory demyelinating disease of the central nervous system (CNS) in young adults. The proinflammatory cytokines such as interferon-gamma (IFN-*γ*), tumor necrosis factor-alpha (TNF-*α*), and nitric oxide (NO) which are known to be produced by inflammatory cells play a key role in the pathogenesis of MS. Some metabolic changes may have an effect on axonal transmission, and white blood cells NO and other inflammatory mediators such as cytokines may be affected from cooling process. In this study, we evaluated the effects of body cooling procedure on proinflammatory cytokines such as TNF-*α*, IFN-*γ*, and NO levels. Twenty patients with MS were evaluated. Thirteen of the patients were women, 7 were men (mean age: 33.6 ± 7.5 yrs.). Body temperature was reduced by an average of 1°C approximately in 1 hour with using the “Medivance Arctic Sun Temperature Management System” device. In our study, the decrease in TNF-*α*, IFN-*γ* levels after the cooling procedure has no statistical significance, whereas the decrease in the mean level of NO level after the cooling procedure is 4.63 ± 7.4 *μ*mol/L which has statistical significance (*P* = 0.002). These results suggested that the decrease in NO level improves conduction block in demyelinated axonal segments after cooling procedure in multiple sclerosis.

## 1. Introduction

Multiple sclerosis (MS) is the most common inflammatory demyelinating disease of the central nervous system (CNS) in young adults. Current knowledge indicates that tissue damage in MS is due to a T-cell-mediated autoimmune process. Once MS has been triggered, a pathogenic autoimmune T-helper −17 subtype of T cells response directed against myelin/CNS components perpetuates the disease [1, 2]. The proinflammatory cytokines such as interferon-gamma (IFN-*γ*), tumor necrosis factor-alpha (TNF-*α*), and nitric oxide (NO) which are known to be produced by inflammatory cells play a key role in the pathogenesis of MS [3–6].

Many individuals diagnosed with MS are sensitive to increased body temperature. The earliest medical reports of thermal sensitivity in MS are derived from Ollivier d'Angers in 1827 [7]. However, Wilhelm Uhthoff's description of this phenomen on occurring after a hot bath or with exercise in MS patients with a history of optic neuritis has most commonly been cited as the landmark observation of the pathophysiological principle of temperature-induced conduction block in demyelinated axonal segments [8]. It is estimated that 60–80% of the MS population experiences transient and temporary worsening of clinical signs and neurological symptoms as a result of elevated body temperature [9–12]. Conversely decreasing body temperature increases the conduction and may cause alleviation of the symptoms [13–19].

It is thought that the improvement observed clinically due to cooling is related to the temperature change in structures adjacent to demyelinated axons [20, 21]. Some metabolic changes may have an effect on axonal transmission, and white blood cells NO levels and other inflammatory mediators such as cytokines may be affected from cooling process. The conduction in demyelinated axons is particularly sensitive to block by NO. The inflammation may directly cause symptoms via nitric oxide release, and its inhibition may lead to the improvement of symptoms [22].

The aim of this study was to investigate the changes of TNF-*α*, IFN-*γ*, and NO level in sera both in normal body temperature and after decreasing body temperature (approximately −1 degree C) in patients with multiple sclerosis.

## 2. Materials and Methods

### 2.1. Patients

From January 2009 to July 2012, 20 patients with clinically definite MS, according to McDonald Criteria (2005), all with previous optic neuritis, were enrolled in the study. This study was conducted by the collaboration of Department of Neurology, Anesthesiology and Reanimation, and Biochemistry in Dokuz Eylül University Hospital, in a multidisciplinary, cross-sectional manner. The study was guided by common ethical principles in research and according to the Declaration of Helsinki. The Regional Ethical Board at the Faculty of Medicine, Dokuz Eylül University, Turkey (July,6,2009/13501), also approved the study. The cooling procedure was explained in detail, and the informed consent was taken from all patients.

Thirteen of the patients were women, 7 men (mean age: 33.6 ± 7.5 yrs, range (20–48 years)). The disease duration was 4.25 ± 3.16 yrs. The number of relapse was 3.25 ± 1.55. Sixteen patients were relapsing remitting multiple sclerosis (RRMS), 4 were secondary progressive multiple sclerosis (SPMS). The mean EDSS score was 3.42 ± 1.60. In all the patients, the disease was in remission period. No patient was affected by any general disease apart from MS. The demographic features of the patients were presented in Table 1.

Exclusion criteria were the presence of fever, infections, or a relapse and the use of corticosteroids within the past 3 months. Patients were excluded if they had used antihypertensive or vasoactive medications or diuretics within the previous month or if they had other significant medical diagnoses including thyroid, hypothalamic, and cardiovascular disease.

All procedures were applied in Neurology Department and the levels of TNF-*α*, IFN-*γ*, and NO were studied in Neuroimmunology Laboratory.

### 2.2. Cooling the Body Procedure

Body temperature was reduced by an average of 1°C approximately in 1 hour with noninvasive, “latex-free, nonirritant hydrogel pads” using the “Medivance Arctic Sun Temperature Management System” device. 

Twelve hours before the procedure, patients started fasting as they do in preanesthesia/presedation. The procedure was performed in a “Neurosensory Neurophysiology Research Laboratory” suitably equipped with an oxygen tube, immobile aspirator, surgery stretcher, and technical equipment to stabilize the bag-mask-valve and vital functions for the implementation of anesthesia. Room temperature was kept stable at 21°C before the procedure. All patients underwent the procedure between 09:00 and 12:00 in the morning. 

Patients wore surgical gowns and colonoscopy shorts before the procedure. 

Four cooling pads were used. Two of them were placed over the thoracoabdominal region. The other two were placed over the femoral region. Later, a highly sensitive rectal probe with a feature of an accurate heat transducer (Mr. Smith Level 1 Esophageal/Rectal Temperature Probe with 400 Series Thermistor) was inserted approximately 6–8 cm into the rectum. All the pads and the probe were secured and then connected to the device. 

Before the procedure, baseline measurements of all the patients (heart rate (HR), systolic arterial blood pressure (SBP), diastolic arterial pressure (DAP), mean arterial pressure (MAP), oxygen saturation (SPO2), respiratory rate, and rectal and oral body temperature) Hewlett Packard, M1094B-Saronno, Italy, were recorded.

All the patients were administered 0.9% saline infusion intravenously with a 20-gauge intravenous cannula inserted into the dorsum of the hand. Patients were delivered oxygen for 2–4 L/minute with a face mask during the cooling procedure. Titrated Midazolam (0.05 mg/kg) and propofol 0,5 mg/kg were administered under the supervision of the anesthesia team. The sedation levels were assessed with the Ramsay Sedation Scale, and the sedation score was arranged as 3-4. 

**Figure 1:**
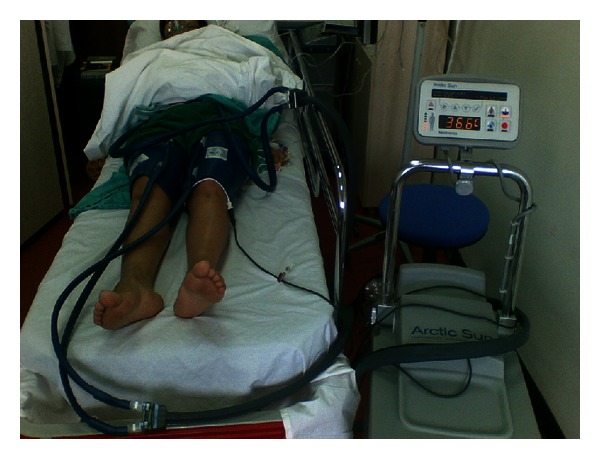
The machine and method used for body cooling.

Sedation took a period of approximately one hour. Rectal body temperature was continuously monitored with the “Medivance Arctic Sun Temperature Management System” device. Oral body temperature was checked every 10 minutes. The device had a feature to drop the temperature of distilled water in the system down to 6°C. Based on the hypothermic response of the body, it cooled the water in the system at a rate to keep vital functions stable. After the rectal body temperature dropped by approximately 1°C, the process was terminated. The machine used for body cooling was shown in Figure 1. 

### 2.3. Cytokines and Nitric Oxide

 All patients' blood was collected at the start of study, and just after lowering the body temperature by 1°C to detect Leukocyte NO_2_ (Nitrate and Nitrite) and Cytokine (TNF-alpha and IFN-gamma). Blood collected in biochemical tubes in vertical position was kept in waiting for 45 minutes and was then centrifuged at 3500 rpm for 15 minutes. Without delay, resulting serum samples were preserved at −80°C until the day of the study. TNF-alpha and IFN-gamma levels were measured by “sandwich” ELISA method, and leukocyte NO level was measured using spectrophotometric method.

Serum specimens were assayed for concentrations of TNF-alpha and IFN-gamma using ELISA kits according to the manufacturer's instructions (Invitrogen Human Immunoassay Kits), for concentration of leukocyte NO using NO Assay Kit (Assay Designs NO Assay Kit) and Cut-off Filters 50 PK (R&D Systems Inc.).

### 2.4. Statistical Analysis

For the statistical analysis, SPSS 15.0 for Windows software is used. Frequency tables are presented for the categorical variables and descriptive statistics (average, standard deviation, median, minimum, maximum) are presented for the numeric variables. In categorical comparison between the groups, cross table statistics are given and significance level is examined through Chi square test. In order to measure the dependence between the groups for which the normal distribution condition is provided, Spearman's rho coefficient is calculated. The comparison of the values before and after the treatment is carried out by using paired-sample *t*-Test. The statistical alpha significance level is accepted as the condition where *P* value is <0,05.

## 3. Results

In our study, 14/20 MS patients (70%) had Uhthoff's phenomenon and all patients (100%) determined improvement in their symptoms (anti-Uhthoff effect) with decreasing environmental temperature. In thirty-five percent of patients (35%), exercise/fatigue and in thirty percent (30%) more than one factor which can create UF has led to worsening of clinical findings. UF is present in 62.5% of RRMS patients and in all of SPMS (100%) patients.

### 3.1. Cooling Procedure

Prior to the cooling procedure, the rectal temperature is determined as average 37.14 ± 0.68°C, oral temperature is determined as average 36.75 ± 0.54°C. Acquiring 1°C decrease in oral temperature, the procedure is terminated. The mean time of cooling procedure was 54.75 ± 11.06 minutes (ranges 40–80 minutes). The rectal temperature after the procedure decreased by 0.89 ± 0.13°C average, the oral temperature decreased by 1.3 ± 0.26°C average. Prior to the procedure, the average oxygen saturation is determined as %99.75 ± 0.63, pulsation 84.6 ± 14.29 throbs, systolic blood pressure 111.25 ± 10.86 mmHg, and diastolic blood pressure 70.75 ± 10.03 mmHg. After the procedure the oxygen saturation decreased %0.25 ± 0.64, pulse rate decreased 7.80 ± 9.95, systolic blood pressure decreased 9.50 ± 6.86 mmHg, and diastolic blood pressure decreased 8.75 ± 7.76 mmHg. The changes of vital functions during the cooling procedure are presented in Table 2.

### 3.2. Nitric Oxide (NO), TNF-Alpha, and IFN-Gamma Levels before and after Cooling Procedure

The average NO level prior to the cooling is determined as 10.96 ± 10.4 *μ*mol/L, average NO level after the cooling is determined as 6.33 ± 7.9 *μ*mol/L. The decrease in NO level before and after the cooling procedure is average 4.63 ± 7.4 *μ*mol/L which has statistical significance (*P* = 0.002) (Table 3). The average TNF-*α*, level prior to the cooling is determined as 6.04 ± 4.4 *μ*mol/L, average TNF-*α* level after the cooling is determined as 5.33 ± 3.2 *μ*mol/L. The decrease in TNF-*α* level before and after the cooling procedure is average 0.71 ± 2.6 *μ*mol/L which has no statistical significance (Table 3). The average IFN-*γ* level prior to the cooling is determined as 11.01 ± 26.4 *μ*mol/L, average IFN-*γ* level after the cooling is determined as 9.25 ± 11.2 *μ*mol/L. The decrease in IFN-*γ* level before and after the cooling procedure is average 1.76 ± 7.23 pg/mL which has no statistical significance (Table 3).

No statistical significance is determined between the differences in mean NO, TNF-*α*, and IFN-*γ* levels by cooling procedure and the application time. No statistically significant relation is determined between the duration multiple sclerosis disease and the number of attacks and differences between the precooling and postcooling mean NO, TNF-*α*, and IFN-*γ* I levels. There is no statistically significant effect of difference in the nitric oxide levels resulting from the cooling procedure over the difference in the mean EDSS scores and functional subgroup scores. 

## 4. Discussion

Multiple sclerosis is an inflammatory demyelinating disorder of the CNS, with symptoms resulting from impaired conduction through demyelinated and transected axons [23]. It has been recognized that the worsening of symptoms that occurs with heating of the body and elevation of its temperature is followed, upon recooling, by a restitution of the symptoms and signs to the state that existed before heating [24]. In MS some symptoms associated with heat exposure and physical exertion are fatigue, general weakness, gait disturbance and exacerbation of most preheat signs and symptoms [11]. Conversely, many patients with MS experience improvement of their symptoms and signs after exposure to cold. A reduction of body temperature of about 1°F is necessary for such improvement [24]. It is thought that the improvement observed clinically due to cooling is related to the temperature change in structures adjacent to demyelinated axons; however, this is not the only explanation [20, 21]. Brenneis et al. (1979) suggested that a noticeable part of neurological deficit is reversible if we were able to raise the threshold of conduction block, which depends on temperature, ph., electrolytes, and neurotransmitters and that the effects of temperature may be associated with multiple factors [25].

The mechanism of the formation of conduction blocks by heating the body is debatable.

In our study, the decrease in IFN-*γ* and TNF-*α* levels before and after the cooling procedure has no statistical significance, whereas the decrease in NO level before and after the cooling procedure is average 4.63 ± 7.4 *μ*mol/L, which has statistical significance (*P* = 0.002). 

Although several mechanisms may be responsible for the beneficial effect of cooling in MS, a lowering of leukocyte NO production may play an important role. Beenakker et al. (2001) studied cooling effect in 10 heat-sensitive MS patients. There was no decrease in tympanic temperature, but cooling procedure was associated with a 41% decrease in mean leukocyte nitric oxide production [26]. This effect on NO could be relevant because it blocks conduction in demyelinated axons [22, 26]. Nitric oxide generated by the inducible form of nitric oxide synthase (iNOS) may contribute to the pathogenesis of multiple sclerosis. iNOS is induced in multiple cell types in MS lesions and astrocyte-derived nitric oxide could be important in coordinating inflammatory responses in MS. Multiple sclerosis is an inflammatory disease of the central nervous system (CNS) that is thought to be mediated by an autoimmune attack directed against components of the myelin sheath. Although the mechanisms that lead to loss of function associated with these immunologically events remain poorly understood. The activation of T cells and macrophages that secrete freely diffusable factors has an important role in the pathogenesis of MS. These factors are the proinflammatory cytokines interleukin IL-1, TNF-*α*, IL-12, and IFN-*γ* and reactive oxygen and reactive nitrogen species. All of these factors have been shown to be elevated in active MS lesions and serum and CSF and animal models support a role for them in disease pathogenesis [27–30]. Nitric oxide is a free radical gas which acts as an important mediator/messenger in neuroprotection, neurotransmission, memory, and synaptic plasticity under physiological conditions [31]. NO has a short half-life and is rapidly converted to more reactive intermediates such as nitrite and nitrate reflecting *in vivo* production of NO [32]. While the demyelination alone is sufficient to block the conduction and thereby cause symptoms, there is increasing evidence that the inflammation may also contribute significantly to the conduction block, although the mechanisms are not understood. Nitric oxide is an important inflammatory mediator which is elevated within the central nervous system in multiple sclerosis and which can be experimentally applied to tissues using nitric oxide donors. NO is a diffusible gas that can enter the CNS and block the conduction in demiyelinated axons through a mechanism that is not completely understood. Smith et al. (1999) suggested that an involvement of NO on ion channel NO has axonal Na + channel modulating properties. NO causes reversible conduction block in both normal and demyelinated axons of the central and peripheral nervous systems [33]. Notably, conduction in demyelinated and early remyelinated axons is particularly sensitive to block by nitric oxide, so that at lower concentrations, including those expected at sites of inflammation, demyelinated axons are selectively affected. Inflammation may directly cause symptoms via nitric oxide release, and the inhibition of such release by cooling may open a new therapeutic avenue for demyelinating disease. 

In our study, active cooling resulted in a significant decrease in leukocyte NO production, and this might provide an explanation for the clinical improvement. Cooling procedure increases the activity of the sympathetic nervous system, which induces an elevation of plasma norepinephrine (NE) [34]. Norepinephrine major sympathetic neurotransmitter. Norepinephrine and dopamine increased lymphocyte activation accompanied by augmented Th1 and Th2 type cytokine production [35]. The activation of the sympathetic nervous system during an immune response may suppress Th1 responses and other products of activated macrophages. NE reduces the release of proinflammatory cytokines by mononuclear cells [36] and this could lead to a reduction in leukocyte NOS activity and NO production [26].

 Chronic fatigue syndrome (CFS) is a disabling illness of unknown etiology. One theory about the etiology of CSF is that it is related to elevated peroxynitrite levels [37]. Like MS, increased levels of inflammatory cytokines, including TNF-*α*, IL-1, IL-6, and IFN-*γ* stimulate the production of elevated levels of iNOS, which in turn synthesizes increased amounts of NO. NO reacts rapidly with superoxide to form peroxynitrite, a potent oxidant. Oxidative stress and NO have been proposed to play an important role in CFS pathophysiology [38]. Suarez et al. (2010) showed important differences in the changes in NO metabolites (nitrates) after exercise in a group of CFS and a control group. They determined that NO metabolites (nitrates) reached the very much higher values in chronic fatigue syndrome patients than normal controls [39].

## 5. Conclusions

Our findings should encourage further research into the role of NO in contributing to the symptomatic manifestations of MS, because this may lead to pharmacologic interventions that mimic the effects of cooling.

## Figures and Tables

**Table 1:** Demographic features of patients with multiple sclerosis.

Subjects (*n*)	20
Gender (F/M) *n*	16/4
Mean age	33,6 ± 7,54
Duration of disease (years)	4,25 ± 3,16
Number of attacks	3,25 ± 1,55
RRMS *n* (%)	16 (80,0)
SPMS *n* (%)	4 (20,0)

F/M: female/male, values are expressed as mean ± SD.

**Table 2:** Pre- and postprocedural changes of body temperature, O_2_ saturation, pulse, and blood pressure.

	Pre-	Postdifference
Rectal temperature (°C)	37,14 ± 0,68	−0,89 ± 0,13
Oral temperature (°C)	36,76 ± 0,55	−1,31 ± 0,26
Oxygen saturation (%)	99,75 ± 0,64	−0,25 ± 0,64
Pulse rate (per min.)	84,60 ± 14,29	−7,80 ± 9,95
Systolic blood pressure (mmHg)	111,25 ± 10,87	−9,50 ± 6,86
Diastolic blood pressure (mmHg)	70,75 ± 10,04	−8,75 ± 7,76

**Table 3:** Pre- and postcooling values of nitric oxide, TNF-*α*, and IFN-*γ*.

	Pre-	Post-	Difference	95% CI	*P*
NO	10,96 ± 10,4	6,33 ± 7,9	4,63 ± 7,4	1,17–8,11	0,002
TNF-*α*	6,04 ± 4,4	5,33 ± 3,2	0,71 ± 2,6	0,52–1,94	0,33
IFN-*γ*	11,01 ± 26,4	9,25 ± 11,2	1,76 ± 7,23	1,64–5,16	0,46

Values are expressed as mean ± SD. Difference: Post: Precooling difference.
